# Investigating the Predictive Validity of the Quantitative Checklist for Autism in Toddlers and the Autism Diagnostic Observation Schedule-2 in Children at Elevated Likelihood for Autism

**DOI:** 10.1007/s10803-024-06585-y

**Published:** 2024-10-13

**Authors:** Sarah Schaubroeck, Ellen Demurie, Jannath Begum-Ali, Sven Bölte, Sofie Boterberg, Jan Buitelaar, Tony Charman, Terje Falck-Ytter, Sabine Hunnius, Mark Johnson, Emily Jones, Greg Pasco, Carlijn Van den Boomen, Petra Warreyn, Herbert Roeyers

**Affiliations:** 1 Research in Developmental Diversity Lab (RIDDL), UGent, Department of Experimental Clinical and Health Psychology, Ghent University, Henri Dunantlaan 2, 9000 Ghent, Belgium; 2 Centre for Brain and Cognitive Development, Birkbeck College, University of London, London, UK; 3 Department of Women’s and Children’s Health, Centre for Neurodevelopmental Disorders, Karolinska Institutet (KIND), Stockholm, Sweden; 4 Department of Cognitive Neuroscience, Donders Institute for Brain, Cognition & Behaviour, Radboud University Medical Centre, Nijmegen, The Netherlands; 5 Department of Psychology, Institute of Psychiatry, Psychology and Neuroscience, King’s College London, London, UK; 6 Department of Psychiatry, Brain Centre Rudolf Magnus, University Medical Centre Utrecht, Utrecht, The Netherlands; 7 Donders Centre for Cognition, Donders Institute for Brain, Cognition & Behaviour, Radboud University, Nijmegen, The Netherlands

**Keywords:** ADOS-2, Q-CHAT, Siblings, Elevated likelihood, Autism, Early screening

## Abstract

This study examined the recurrence rate of autism in siblings at elevated likelihood (EL) and the predictive validity of the Q-CHAT and ADOS-2 at 14 and 24 months (m) for a clinical best estimate (CBE) autism diagnosis at 3 years. 331 EL-siblings (47.9% girls) from the prospective longitudinal EuroSibs study underwent ADOS-2 assessments and caregivers completed the Q-CHAT at 14 m and 24 m. At 3 years CBE was determined using DSM-5 criteria. Sensitivity, specificity, positive predictive value (PPV) and negative predictive value (NPV) were estimated. Autism recurrence rate was 25.7% [95% CI (21.1, 30.6)]. Q-CHAT sensitivity was 31.8% [95% CI (21.4, 43.6)] at 14 m and 30.6% [95% CI (20.7, 41.7)] at 24 m. Specificity was 81.2% [95% CI (75.4, 86.2)] at 14 m and 94.8% [95% CI (91.2, 97.2)] at 24 m. PPV was 35.6% [95% CI (24.2, 48.2)] at 14 m and 66.7% [95% CI (49.8, 81.1)] at 24 m. NPV was 78.5% [95% CI (72.6, 83.7)] and 79.9% [95% CI (74.7, 84.6)] respectively. ADOS-2 demonstrated a of 64.3% [95% CI (45.9, 80.2)] and 69.3% [95% CI (58.4, 79.0)] and a specificity of 71.1% [95% CI (60.3, 80.4)] and 68.7% [95% CI (62.5, 74.5)] at 14 m and 24 m respectively. PPV was 45% [95% CI (30.3, 60.4)] at 14 m and 41.9% [95% CI (33.5, 50.7)] at 24 m. NPV was 84.4% [95% CI (74.2, 91.8)] at 14 m and 87.3% [95% CI (81.9, 91.6)] at 24 m. Q-CHAT and ADOS-2 at 14 m and 24 m can aid in early differentiation between EL-siblings who need further assessment and those who do not, but neither has sufficient sensitivity and PPV for standalone CBE diagnosis prediction.

## Introduction

Autism spectrum disorder (henceforth autism) is a neurodevelopmental condition, which emerges in childhood and is characterised by difficulties in two main areas: challenges in social communication and social interaction alongside restricted, and repetitive patterns of behaviour, interests, or activities and sensory processing differences that cause problems to comply with society’s demands and expectancies (diagnostic and statistical manual of mental disorders (DSM-5-TR); American Psychiatric Association [APA], 2022). The estimated global prevalence is around 1% (Zeidan et al., 2022). However, significant regional variations exist, with recent U.S. studies reporting rates as high as 2% in the general population (Maenner et al., 2023). These regional differences may reflect varying diagnostic practices, increased awareness, or other socio-environmental factors. Additionally, children with an older sibling with autism have a significantly elevated likelihood (EL) to have autism. Research focused on siblings suggests that approximately one in ten to one in five siblings of children diagnosed with autism receives an autism diagnosis by the age of 3 years. Overall this represents a 20-fold increase in likelihood of autism compared to the general population (Messinger et al., 2015a, 2015b; Ozonoff et al., 2011, 2024). This strong heritability is supported by large-scale population studies, where the recurrence rate of autism among siblings is found to be around 10% (Hansen et al., 2019; Jokiranta-Olkoniemi et al., 2016; Sandin et al., 2017).

A meta-analysis of 35 studies from van’t Hof et al. (2021), including 55 cohorts from 35 countries and involving 66,966 individuals with autism, found a current mean age at diagnosis of 60.48 months (range: 30.90–234.57 months). In a subgroup analysis of studies that included only children aged 10 years or younger (nine studies, 26 cohorts from 23 countries, n = 18,134 children with autism), the mean age at diagnosis was 43.18 months (range: 30.90–74.70 months) (van’t Hof et al., 2021). However, developmental vulnerabilities associated with autism are often already identifiable with parent report screeners in toddlers from 12 to 18 months of age (Canu et al., 2021; Chlebowski et al., 2013; Pierce et al., 2011; Robins et al., 2014a, 2014b; Sacrey et al., 2021; Turner-Brown et al., 2013). For siblings of children diagnosed with autism, but also for children who show atypical developmental patterns, an earlier diagnosis would be beneficial. Early identification of autism allows for timely guidance or intervention, potentially reducing negative outcomes associated with autism. Moreover, early intervention might have the potential to limit the occurrence of secondary problems and lead to a more optimal developmental trajectory and improved quality of life (Fuller & Kaiser, 2020; Ryberg, 2015; Towle et al., 2020).

Nevertheless, early diagnosis of autism poses a multifaceted challenge. Several studies describe children with subthreshold diagnostic features of autism who may have an increased susceptibility to gain or lose autism features at later ages (Charman et al., 2017; Tunç et al., 2021). Tunç et al. (2021) highlight the inherently dynamic nature of early autism, contributing to the complexity of attaining an early and accurate diagnosis as some children may not display clear autism features nor clear typically developing profiles. This intermediate phenotype is one of the factors contributing to the complexity of attaining an early and accurate diagnosis. Another significant challenge arises from the phenotypic heterogeneity often observed in siblings, which includes co-occurring difficulties or conditions such as (features of) attention deficit hyperactivity disorder (ADHD), developmental delay, or language delay (Charman et al., 2023; Hus & Segal, 2021; Shephard et al., 2017). This variability highlights the importance of diagnostic tools with strong prospective validity to effectively distinguish autism from other conditions. However, it’s important to consider that early diagnosis also carries certain risks, including over- or misdiagnosis, causing unnecessary parental distress and additional strain on already limited healthcare resources.

Consequently, it is important to evaluate the psychometric properties of screening and diagnostic instruments commonly used to assess autism features. Currently, there is no single measure that is recommended for the early detection of autism (Fuentes et al., 2021) and screening practice guidelines vary widely both within and across countries (DeLucia et al., 2022). While several autism screening tools are widely accessible (Petrocchi et al., 2020), their psychometric properties strongly depend on the sample and clinical expertise remains crucial in the diagnostic process (Bishop & Lord, 2023; Charman & Gotham, 2013). A recent systematic review (Levy et al., 2020) indicates that widely used autism screening tools, when applied to general pediatric populations aged 16–40 months, can accurately identify many children with autism. However, the psychometric properties vary significantly across different age groups, settings, and contexts. For example, one of the most extensively studied tools, the M-CHAT, has shown predictive values (PPVs) ranging from 0.06 to 0.60 (Baduel et al., 2017; Robins et al., 2014a, 2014b; Sturner et al., 2016). In general, sensitivity (indicated by the proportion of children with autism who also have an instrument positive result, i.e. true positives) is higher than specificity (indicated by the proportion of children without autism who have an instrument negative result, i.e. true negatives) (Charman et al., 2007, 2016; Fuentes et al., 2021; Raza et al., 2019), although some studies have reported contrasting findings (Allison et al., 2021; Guthrie et al., 2019; Robins et al., 2014a, 2014b). While sensitivity and specificity are informative about the diagnostic validity of an instrument, other indices are prognostic and tell us something about the individual child, such as the positive predictive value (PPV; i.e., the probability that a child with a positive test result actually has autism) and negative predictive value (NPV; i.e., the probability that a child with a negative test result does not have autism). These values vary widely between studies, depending on the prevalence rate of autism and the setting where screening is occurring (Dawson et al., 2023; DeLucia et al., 2022; Levy et al., 2020). In the general population, where the prevalence of autism is about 1%, the positive predictive value is generally low, while the negative predictive value tends to be higher (Guthrie et al., 2019). Nevertheless, studies that report estimates of sensitivity, specificity, and predictive value are limited, as systematic follow-up of all children, including those who test negative, is necessary to calculate these measures (Petrocchi et al., 2020). Unfortunately, follow-up of children with negative results is often lacking, making it challenging to evaluate these assessment measures.

Allison et al. (2021) investigated the ability of the Quantitative Checklist for Autism in Toddlers (Q-CHAT; Allison et al., 2008) to identify autism in young children between 18 and 30 months in the general population. They reported an Area Under the Curve (AUC) value of 0.85 and, using a cut-off of ≥ 39, they found poor sensitivity (0.44) and excellent specificity (0.98) rates. The PPV was poor (0.28), while the NPV was almost perfect (0.99). Similar research on the psychometric properties of screeners and diagnostic instruments in EL-siblings is scarce. Given the elevated base rate, the abovementioned estimates will differ from the general population, impacting the usefulness of autism screeners in the EL-population (Wieckowski et al., 2023). There is a potential for a higher PPV, which could enhance the classification accuracy of screening methods. Raza et al. (2019) report fair sensitivity rates (0.75 at 18 months and 0.71 at 24 months), but poor specificity (0.63 at 18 months and 0.65 at 24 months) for the Short Quantitative Checklist for Autism in Toddlers (Q-CHAT-10; Allison et al., 2012) in an EL-sibling cohort. The PPV was low (0.36 and 0.34 at 18 and 24 months, respectively), but NPV was high (0.90 at both ages) in relation to the diagnostic assessment at 3 years old. While the sensitivity and PPV are slightly higher, these results indicate that the utility of the Q-CHAT-10 solely for clinical applications is limited, as there might be an overidentification of toddlers who will eventually not be diagnosed with autism (i.e., high rate of false positive screens) (Raza et al., 2019). To our knowledge, the psychometric properties for the full Q-CHAT in EL-siblings has not yet been investigated.

In clinical practice, the autism diagnostic observation schedule-2 (ADOS-2: Lord et al., 2012b) is one of the most widely used diagnostic instruments for autism assessment. A recent review, in children between 9 months and 14 years old, indicated high levels of sensitivity (ranging from 0.89 to 0.92) and specificity (ranging from 0.81 to 0.85) for the ADOS-2, mostly in clinically referred cohorts (Lebersfeld et al., 2021). However, in EL-siblings specifically, Zwaigenbaum et al. (2016) found at 14 and 24 months a rather low cross-sectional agreement between ADOS-2 classification and clinical best estimate (CBE) diagnosis. The discrepancy may lie in the fact that the review focused on pre-screened or referred children, while the study of Zwaigenbaum et al. (2016) involved only siblings of children with autism. Additionally, the early age (14 and 24 months) at which the CBE was conducted in the paper by Zwaigenbaum et al. (2016) may also contribute to the lower agreement. The predictive accuracy of ADOS-2 in EL-siblings prior to the age of 3 years is unclear and the PPV and NPV of diagnostic instruments such as the ADOS-2 are often not reported for this age-group. Not only should sensitivity and specificity be taken into account, but also the PPV of children with confirmed diagnoses and NPV of children with an initial negative screen or non-autism classification.

In short, it is important to continue to extend our knowledge concerning which commonly used screening and diagnostic instruments can detect and predict autism characteristics at an early age. Younger siblings of autistic individuals have an elevated likelihood to also develop autism and are a substantial group within the total autism population, but to date, there is limited data available regarding the psychometric properties of screening and diagnostic instruments in EL-siblings.

### The Present Study

The present study evaluated the predictive validity of the Q-chat and the ADOS-2 in EL-sibling toddlers. In order to do this, it is important to determine the recurrence rate of autism in our EL-sibling sample. Therefore, our first aim was to estimate the autism recurrence rate in a large European sibling cohort. We expected similar prevalence rates at the age of three as found in non-European yet Western samples, based on DSM-IV-TR (Ozonoff et al., 2011). Second, this study aimed to evaluate the predictive validity of an early screening tool for detecting autism characteristics in EL-siblings, the Quantitative Checklist for autism (Q-CHAT; Allison et al., 2008), at 14 and 24 months. As there seems to be a gap between the age of detection of early signs of autism using screeners and the age of actual diagnosis (Pierce et al., 2019; van’t Hof et al., 2021), it is important to examine how accurately these screeners can predict diagnostic outcome in EL-siblings. Although previous research on other screeners in EL-siblings sometimes found higher sensitivity than specificity (Charman et al., 2007; Duvekot et al., 2015; Raza et al., 2019), we had no clear predictions for the Q-CHAT. Additionally, we did expect PPV to be higher in EL-siblings compared to children with a typical likelihood for autism. Third, we examined the predictive validity of the most used diagnostic instrument for autism, the ADOS-2 (Lord et al., 2012b) in EL-siblings at the ages of 14 and 24 months. Our aim was to build on the limited information on sensitivity, specificity, positive and negative predictive value of the ADOS-2 in EL-siblings. Based on previous literature (Ozonoff et al., 2015; Zwaigenbaum et al., 2016), we expected sensitivity to be high, but specificity to be poor, indicating few false negatives, but a substantial number of false positives. In line with Zwaigenbaum et al. (2016) we expected that the positive predictive value and negative predictive value would increase with age.

## Methods

### Participants

The sample for this study was drawn from a prospective, longitudinal study conducted by the EuroSibs Autism Research Network (Jones et al., 2019) in which a cohort of siblings at EL for autism was assessed at the ages of 5, 10, 14, 24 and 36 months. Ethical approval was given by local ethics committees in participating countries and the study was conducted in accordance with the Declaration of Helsinki and the American Psychological Association. Parents gave written informed consent. The EL-group consisted of children who had at least one older sibling with a community clinical diagnosis of autism. Families of EL-infants were recruited through various channels, including well-baby clinics, child care centers and volunteer databases. Additionally, EL-families were specifically recruited through diagnostic and intervention services for autism and events for parents of children diagnosed with autism. Exclusion criteria for EL-infants included diagnosis of epilepsy, preterm birth (i.e. < 36 weeks of gestational age), and genetic syndromes clearly related to autism. For the current study, data from an EL cohort at four sites were used: the Early Autism Sweden (EASE) in Sweden (n = 221), The Babystudy project in Belgium (n = 60), the Siblings of Children with Autism (ZEBRA) project in the Netherlands (n = 52), and the Studying Autism and ADHD in the eaRly yearS (STAARS) project in the UK (n = 101). Participants were excluded if there was no Clinical Best Estimate (CBE) available (n = 103). This resulted in a final sample of 331 EL-siblings (47.9% girls) distributed as follows: Sweden (n = 158), Belgium (n = 52), the Netherlands (n = 40), UK (n = 81). Child characteristics of the final sample are shown in Table 1.

**Table 1:** EL-sibling characteristics

	CBE autism n = 85		CBE non-autism n = 246	
	Frequency (%)		Frequency (%)	
Sex (female:male)	33:52 (38.85:61.15)		124:122 (50.6:49.4)	
Maternal education				
1	3 (4.48)		4 (1.97)	
2	21 (31.34)		49 (24.14)	
3	19 (28.36)		67 (33.00)	
4	24 (35.82)		83 (40.89)	
Missing	18		43	

	Mean (SD)	Range	Mean (SD)	Range
Age (months)				
14 month visit	14.34 (.52)	12.98–16.95	14.40 (.66)	12.98–16.95
24 month visit	24.82 (1.07)	23.20–29.47	24.92 (1.21)	23.16–30.63
36 month visit	38.27 (2.75)	35.57–47.41	37.84 (2.25)	35.53–48.79
ELC (MSEL)				
14 month visit	86.44 (13.68)		91.63 (14.99)	
24 month visit	85.25 (19.08)		99.48 (16.50)	
36 month visit	88.52 (23.72)		105.16 (17.44)	

*SD* standard deviation, *CBE* clinical best estimate, *Maternal education* highest achieved diploma, 1 primary education, 2 secondary education, 3 tertiary-undergraduate, 4 tertiary-postgraduate, *ELC* early learning composite, *MSEL* Mullen scales of early learning

### Procedure

For this study, data collected during the 14-, 24-, and 36 month visits of the cohort studies were utilized. At each assessment time point, participants underwent a comprehensive battery of standardized assessments, including an evaluation of the child’s language and cognitive development using the Mullen scales of early learning (MSEL; Mullen, 1995). Autism characteristics were assessed with the ADOS-2 at both ages (Lord et al., 2012b). Additionally, parents were asked to fill out a form that included several sociodemographic questions together with the screening questionnaire Q-CHAT (Allison et al., 2008) at 14 and 24 months. Researchers were blind to the Q-CHAT scores when they performed the observational tasks. The children were categorized in two groups by means of their CBE diagnostic outcome. As such, 85 children qualified for the diagnostic criteria of autism according to the DSM-5 (APA, 2013) and 246 children were categorized as non-autistic. The reasons of missingness across time points and instruments include missed appointments or instruments not administered (e.g. ADOS-2 at 14 months was not administered in the UK and missing in most of the children from Sweden). Missing data were not imputed, as imputation methods were deemed inappropriate due to the nature of the aims. Therefore, the reported analyses are based on the available data per instrument.

### Measures

#### Autism Characteristics

##### Autism Diagnostic Observation Schedule-2

The ADOS-2 (Lord et al., 2012b) is a standardized, semi-structured, play-based clinician administered observational measure designed to assess autism features related to communication, social interaction, play, and restricted or repetitive behaviours and interests. The ADOS-2 consists of different modules, with module selection based on chronological age and language ability at time of assessment. In the present study, either the Toddler module (ADOS-T), Module 1 or Module 2 was administered, see Table 2 for an overview. The described guidelines for module selection of the ADOS-2 were followed. All observations were performed by ADOS-2 trained researchers, who had met the research requirements of standardized administrations and scoring reliability. Each module is scored using a diagnostic algorithm that incorporates items from the domains of social affect (SA), which includes communication and social items, and restricted repetitive behaviours (RRB). Across all modules, an ADOS-2 calibrated severity score (CSS) is derived, with a possible range of 1–10, with the threshold for an autism diagnosis being 4 or higher (Gotham et al., 2009). The Toddler Module CSS reported in the current study was calculated based on Esler et al. (2015). The Module 1 and 2 CSS was calculated based on Hus et al. (2014). The ADOS-2 has shown consistently good Cronbach’s alphas in the SA domain (0.87–0.92), while Cronbach’s alphas in the RRB domain ranged from poor (0.51) to questionable (0.66) (Lord et al., 2012a, 2012b). The ADOS-2 has shown good interrater reliability across modules (intraclass correlation coefficients; SA domain = 0.96, RRB domain = 0.84 and total score = 0.96; Lord et al., 2012a, 2012b).

**Table 2:** ADOS-2 modules and classifications per age

Age	Module (ADOS-2 classification)		
	Total frequency (% of different classifications)		
	Toddler module (CSS ≥ 4)	Module 1 (CSS ≥ 4)	Module 2 (CSS ≥ 4)
14m	103 (38.8%)	NA	0
24m	281 (39.1%)	NA	24 (58.3%)

##### The Quantitative Checklist for Autism in Toddlers (Q-CHAT)

The quantitative checklist for autism in toddlers (Q-CHAT; Allison et al., 2008) is a parent-report questionnaire designed to screen for autism-related traits in toddlers aged 14–24 months. It consists of 25 items where parents are asked to rate their child’s behaviours using a 5-point Likert scale, ranging from 0 (e.g., ‘not at all’) to 4 (e.g., ‘always’). Total Q-CHAT scores can range from 0 to 100, with higher scores indicating a greater number of autism-related traits. A total score ≥ 39 is considered as a positive screen (Allison et al., 2012). We have investigated whether we could lower the cut-off to increase sensitivity, but this would significantly reduce specificity, with little improvement in sensitivity. It is not feasible to achieve acceptable levels for both parameters simultaneously. The Q-CHAT has shown adequate to good internal consistency (Cronbach’s alpha of 0.67 and 0.83 in the general population vs. autism group respectively; Allison et al., 2008).

#### Child Developmental Characteristics

Mullen Sales of Early Learning. Children’s verbal and non-verbal skills were assessed using the Mullen scales of early learning (MSEL; Mullen, 1995). The MSEL is a standardized developmental test designed for children aged 1–68 months. In this study, four domains were assessed: fine motor skills, visual perception, receptive language, and expressive language. For each domain, raw scores were converted into T-scores and percentiles to allow for standardized comparisons. To represent the child’s overall cognitive ability, an Early Learning Composite (ELC; M = 100, SD = 15) was derived by summing the T-scores of the assessed domains. The MSEL were administered by trained researchers who followed strict guidelines, including completing role-playing, scoring videos, and achieving 90% scoring reliability across three supervised assessments. An experienced clinician or researcher reviewed and validated all training assessments. Documentation of training and qualifications was monitored by the Clinical Data Monitoring committee. The MSEL has shown good internal consistency (Cronbach’s alpha’s ranging from 0.75 to 0.85 across all scales) and interrater reliability (intraclass correlation coefficients ranging from 0.91 to 0.99; Mullen, 1995). Results from the MSEL are reported in Table 1.

#### Clinical Best Estimate Diagnosis (CBE)

The CBE outcome was defined by the diagnostic criteria of DSM-5 (APA, 2013), following assessment at 36 months. The diagnostic decision was based on clinical professional consensus judgment, which is considered to be the gold standard for diagnosing autism (Klin et al., 2000; Ventola et al., 2006). The decision was not only based on whether or not children scored above the cut-off for autism on the ADOS-2, the diagnostic parent interview (i.e., ADI-R) and parent reported screeners, but was also informed by item level or qualitative information of these instruments, the ELC and subscale scores of the MSEL, developmental assessment administrations, clinical observations made by the researchers during the assessments and all other available information. All diagnoses were established by a team of at least one clinical psychologist with specialized experience in assessing young children with developmental disorders within a multidisciplinary clinical setting together with at least one researcher who had been present during the assessments of the children and who had either extensive clinical or research experience with young children with autism.

#### Maternal Education

Maternal education was assessed but was not included in main analyses. In a questionnaire, we collected information regarding the mother’s highest level of education, which was coded into a numeric score. A score of one refers to primary education only (the equivalent of primary or middle school), score two refers to secondary education (the equivalent of high school), score three was assigned to mothers who finished tertiary education or education at undergraduate level (the equivalent of non-university higher education or a bachelor’s degree) and score four refers to university-level higher education (master’s degree or higher). Maternal education level was included as an approximate measure of social economic status (SES) (Duncan & Magnuson, 2005). However, we are aware that this indicator of SES only reflects a part of this broad construct. Descriptives on maternal education are reported in Tabl﻿e 1.

### Data Analysis

First, descriptive analyses were used to examine the prevalence of autism in this sample of EL-siblings. A 95% binomial proportion confidence interval (CI) was calculated using the Wilson score method (Newcombe, 1995). A binomial logistic regression was performed to ascertain the effects of sex, age, site and maternal education on the likelihood of receiving a CBE of autism. Linearity of age with respect to the logit of CBE outcome was confirmed via the Box and Tidwell procedure (Box & Tidwell, 1962).

To address the second research aim concerning the predictive validity of the Q-CHAT for determining CBE diagnosis of autism, we first explored the relationship between the instrument scores and diagnostic status. Because the data were not normally distributed, we employed Mann–Whitney U tests to examine whether the Q-CHAT scores differed significantly between children with and without a CBE diagnosis of autism. Mean ranks are compared, since distributions of participant characteristic variables were not similar for all groups, which is a requirement for comparing means. Next, descriptive analyses provided us with descriptive information about the Q-CHAT scores at 14 and 24 months, and the agreement between the classifications made by the Q-CHAT scores at 14 and 24 months and the CBE diagnosis of autism at 36 months was evaluated. To measure these pair-wise agreements, Cohen’s kappa next to the sensitivity, specificity, positive predictive value (PPV) and negative predictive value (NPV) was calculated. The overall agreement (corrected for change) using Cohen’s kappa was interpreted according to the following guidelines: < 0 = poor; 0.0–0.20 = slight; 0.21–0.40 = fair; 0.41–0.60 = moderate; 0.61–0.80 = substantial; 0.81–1.00 = almost perfect (Cohen, 1960). To assess the overall accuracy and discriminative power of the Q-CHAT without relying on specific cut-offs, we calculated Receiver Operating Characteristic (ROC) AUC. This value was interpreted according to the guidelines of Hosmer et al. (2013): 0.50 = no discrimination; 0.51–0.69: poor discrimination; 0.70–0.79 = acceptable discrimination; 0.80–0.89 = excellent discrimination; ≥ 90 = outstanding discrimination. The guidelines proposed by Glascoe (2005) were used to interpret the screening and diagnostic psychometric parameters (sensitivity and specificity): < 70% = poor; 70–79% = fair; 80–89% = good; 90–100% = excellent. The Wilson score method (Newcombe, 1995) was again used to calculate 95% CI’s.

Regarding the third research aim, we employed a series of Mann–Whitney U tests to examine whether the ADOS-2 CSS scores at 14 and 24 months differed significantly between children with and without a CBE diagnosis of autism. The agreement between the classification made by the ADOS-2 scores and the CBE diagnosis at 36 months was evaluated using Cohen’s kappa. Furthermore, ROC AUC was also calculated to assess the overall accuracy of the ADOS-2 next to sensitivity, specificity, positive predictive value (PPV) and negative predictive value (NPV). The Statistical Package for the Social Science Software version 28 (IBM Corp, 2021) was used for data-analysis.

## Results

### Aim 1: Estimation of the Autism Prevalence Rate in EL-Siblings

Of the sample of 331 EL-siblings, 85 [25.7%; 95% CI (21.1, 30.6)] received a CBE diagnosis of autism at 36 months of age based on DSM-5 criteria. The remaining 246 EL-siblings were classified as non-autistic. Among those diagnosed with autism, 52 were boys (58.8%) and 33 were girls (41.2%). The logistic regression model was not statistically significant χ^2^(5) = 5.906, p = 0.316). This indicates that the combined predictors did not significantly explain the likelihood of having a CBE diagnosis of autism (Nagelkerke R = 0.03)^2^. However, the effect of sex was significant (B = − 0.59, p = 0.049).

### Aim 2: Validity of the Q-CHAT

#### CBE Group Differences on the Q-CHAT

We first explored the relationship between the Q-CHAT and diagnostic status. As shown in Table 3, the Q-CHAT score at 14 and 24 months was higher in children with a CBE of autism at 36 months compared to children without autism (14m: U = 5203, p = 0.007; 24m: U = 4405.5, p < 0.001).

**Table 3:** Difference in instrument scores between children with and without a CBE diagnosis of autism at 36 months

Instrument	CBE autism (n = 85)			CBE non-autism (n = 246)			p-Value
	Mean Rank (IQR)	Median	Range	Mean Rank (IQR)	Median	Range	
Q-CHAT 14m	156.67 (2)	33	16–68	127.26 (14)	29	9–62	**.007**
Q-CHAT 24m	185.31 (21)	31	5–61	126.48 (12.25)	21	5–50	** < .001**
ADOS CSS 14m	68.13 (3)	5	1–8	45.98 (2)	3	1–8	** < .001**
ADOS CSS 24m	204.47 (5)	5	0–9	136.22 (3)	2	0–9	** < .001**

*CBE* clinical best estimate, *IQR* interquartile range, *14m* timepoint at 14 months, *24m* timepoint at 24 months, *Q-CHAT* quantitative checklist for autism in toddlers, *ADOS-2 CSS* calibrated severity score of the autism diagnostic observation schedule-second edition

Significant differences are marked in bold

#### Psychometric Properties of the Q-CHAT at 14 and 24 Months

When defining a positive screen on the Q-CHAT as a total score equal or above 39 (Allison et al., 2012), 59 out of the 268 children [22%; 95% CI (17.2, 27.5)] screened positive at 14 months. Based on Cohen’s Kappa, there was a slight agreement between the Q-CHAT identification at 14 months and diagnostic classification based on CBE at 36 months [κ = 0.135, p = 0.027; 95% CI (0.008, 0.252)]. At 24 months, 33 out of 282 screened positive [11.7%; 95% CI (8.2, 16)]. Cohen’s Kappa showed a fair agreement between the Q-CHAT identification at 24 months and CBE diagnosis at 36 months [κ = 0.308, *p* < 0.001; 95% CI (0.183, 0.433)].

Table 4 provides the psychometric properties of the Q-CHAT at 14 and 24 months. At 14 months, the AUC value was 0.61, 95% CI (0.533, 0.687), which is a poor level of discrimination according to Hosmer et al. (2013). Results showed a poor sensitivity and a good specificity. The PPV was poor, while NPV was fair.

**Table 4:** Sensitivity, specificity, PPV and NPV of the different autism assessment instruments as compared to CBE diagnosis based on DSM-5 criteria

Instrument	No. of positive cases		No. of negative cases		Sensitivity	Specificity	PPV	NPV
	True positive	False positive	True negative	False negative	(95% CI)	(95% CI)	(95% CI)	(95% CI)
Screening instruments								
Q-CHAT 14m^a^	21	38	164	45	31.8% (21.4–43.6)	81.2% (75.4–86.2)	35.6% (24.2–48.2)	78.5% (72.6–83.7)
Q-CHAT 24m^a^	22	11	199	50	30.6% (20.7–41.7)	94.8% (91.2–97.2)	66.7% (49.8–81.1)	79.9% (74.7–84.6)
Diagnostic instrument								
ADOS 14m^b^	18	22	54	10	64.3% (45.9–80.2)	71.1% (60.3–80.4)	45% (30.3–60.4)	84.4 (74.2–91.8)
ADOS 24m^b^	52	72	158	23	69.3% (58.4–79)	68.7% (62.5–74.5)	41.9% (33.5–50.7)	87.3% (81.9–91.6)

*PPV* positive predictive value, *NPV* negative predictive value, *CBE* clinical best estimate, *DSM-5* 5th edition of the diagnostic and statistical manual of mental disorders, No. total number, *14m* timepoint at 14 months, *24m* timepoint at 24 months, *36m* timepoint at 36 months, *Q-CHAT* quantitative checklist for autism in toddlers

^a^A Q-CHAT autism positive screen requires meeting or exceeding the cut-point of 39 (Allison et al., 2021)

^b^An ADOS-2 classification of autism requires meeting or exceeding the autism spectrum cut-point. The cut-point for the Toddler Module is a total score ≥ 10 for children between 10 and 20 months old or between 21 and 30 months with little-to-no words or ≥ 8 for children between 21 and 30 months with some words. The cut-point for Module 1 is a total score of ≥ 11 (no-to-few words) or ≥ 8 (some words). The cut-point for Module 2 is a total score of ≥ 7

At 24 months, the AUC value was 0.71 95% CI (0.634, 0.783), which is an acceptable level of discrimination according to Hosmer et al. (2013). Results showed a poor sensitivity, while specificity was excellent. Comparable to 14 months, the Q-CHAT did not identify a substantial proportion of children who received a CBE of autism, however only 5.2% of the children were incorrectly identified at 24 months of age. The PPV was acceptable and the NPV was fair.

### Aim 3: Validity of the ADOS-2

#### CBE Group Differences on the ADOS-2

Mann–Whitney U tests showed that children with a later CBE of autism differed significantly from children without a later CBE of autism with respect to ADOS CSS scores at 14 and 24 months (respectively, U = 598.5, p < 0.001; U = 4764.5, p < 0.001). See Table 3.

#### Psychometric Properties of the ADOS-2 at 14 and 24 Months

Based on Cohen’s kappa, the ADOS-2 classification at 14 and 24 months was significantly associated with diagnostic classification based on CBE at 36 months. The overall agreement was fair at all time points (14 and 24 months; κ = 0.311 p = 0.001; 95% CI (0.248, 0.497) and κ = 0.312 p = 0.001; 95% CI (0.208, 0.416) respectively).

Table 4 provides the psychometric properties of the ADOS-2 at 14 and 24 months. At 14 months the AUC was 0.72 95% CI (0.596, 0.834), which is an acceptable level of discrimination according to (Hosmer et al., 2013). Sensitivity was poor, while specificity was fair. There were approximately 30% false positives. This resulted in a good NPV. The PPV was poor, however, indicating a very low probability that a child with an ADOS-positive result actually received a CBE of autism.

At 24 months the AUC was 0.77 95% CI (0.656, 0.792), which is considered an acceptable level of discrimination. Both sensitivity and specificity were poor. Only 30.7% of the children who received a CBE diagnosis of autism were correctly identified and 31.3% of the children without CBE of autism were correctly excluded. Both NPV and PPV were comparable with the results at 14m. The NPV was good, but, in contrast, the PPV was poor.

## Discussion

This study pursued three aims: (1) assessing autism prevalence in an EL-sibling cohort at age 3, (2) evaluating the predictive validity of the Q-CHAT at 14 and 24 months, and (3) examining the predictive validity of the ADOS-2 in a sample of young EL-siblings.

### Autism Prevalence Rate According to CBE Diagnosis

Our investigation into the prevalence of autism in an EL-sibling cohort at 3 years involved a clinical evaluation according to DSM-5 criteria, employing a follow-up protocol that incorporated parent-reported screening instruments and an observational diagnostic measure (ADOS-2). Based on this comprehensive assessment, we observed an autism recurrence rate of 25.7%. This is in line with rates found in previous research in non-European samples (Messinger et al., 2015a, 2015b; Ozonoff et al., 2011). There was a trend towards an uneven distribution of sex differences in the group with versus without a CBE diagnosis of autism. This contrasts with earlier research (Zeidan et al., 2022), which typically reports significant sex differences in autism diagnoses. However, the observed difference in our study was very small, seemingly due to a higher percentage of boys than girls in the autism group. In line with Ozonoff et al. (2011) we did not find significant differences in maternal education between siblings with or without CBE of autism.

### The Validity of the Q-CHAT

The second aim focused on the predictive validity of the Q-CHAT in identifying autism characteristics in EL-siblings. We found significant differences in Q-CHAT scores between children with and without CBE outcome of autism established at 36 months, which suggests that the Q-CHAT could be considered as a preliminary screening measure for detecting early signs of autism. At 14 months, the Q-CHAT demonstrated poor sensitivity, suggesting challenges in early detection of EL-children with a later CBE diagnosis, but good specificity, indicating a good ability to correctly identify EL-children without a CBE of autism. Direct comparison with previous research is difficult since our study is the first to examine the psychometric properties of the Q-CHAT at such a young age. The PPV was poor, emphasizing a high rate of false positives, while the NPV was fair, showing a reasonable rate of correct rejections. These findings suggest that although the Q-CHAT demonstrates some capability in identifying the absence of concerns, its utility in distinguishing between EL-siblings who will later receive an autism diagnosis and those who will not, or in providing guidance on whether to refer for further autism assessment at this early age, is limited. The low sensitivity may be attributed to the poor discriminative accuracy of the Q-CHAT at this young age, but it is also plausible that certain features may not fully manifest or be easily discernible in very young children. Therefore these subtle differences may elude detection during early assessments. Furthermore, it is plausible that behavioral patterns associated with autism become more apparent as children grow older, making them more identifiable during later assessments. (Landa et al., 2022; Ozonoff et al., 2014; Pierce et al., 2019). Thus, despite the effectiveness of the Q-CHAT in screening for autism traits, its ability to detect these characteristics in very young children, where such features may be less pronounced or identifiable, could contribute to the observed lower sensitivity. Furthermore, the Q-CHAT is completed by parents. Parental reports in EL-siblings may be biased: on the one hand, parents may compare their child to an older diagnosed sibling who displays clear autism characteristics, potentially leading to underreporting. On the other hand, parents may be hyperalert, anxious and prone to notice autism characteristics, which may increase the false positive rate (Herlihy et al., 2015; Ozonoff et al., 2009; Zwaigenbaum et al., 2007).

Some improvement in specificity was observed at 24 months, which aligned with findings from the study conducted by Allison et al. (2021). However, sensitivity remained poor, notably lower compared to the rates reported in the study of Allison et al. (2021). The PPV increased to 66.7%, indicating a moderate ability to correctly identify EL children with a CBE of autism, while NPV remained stable. Compared to the Q-CHAT performance in the general population (PPV: 28%; Allison et al., 2021), the PPV was slightly better, which is not surprising given that the prevalence is higher. However, when considering the low sensitivity alongside these findings, the clinical utility of the Q-CHAT at 24 months remains insufficient. Based on these results, reliance on the Q-CHAT for diagnostic referral would result in under-identification of children who will most likely receive an autism diagnosis at a later age, leading to a high false negative rate. Autism characteristics becoming more evident from 24 months onwards (Ozonoff et al., 2015; Zwaigenbaum et al., 2016) may contribute to a rising PPV. Nevertheless, the false positive rate is still considerably high. Parents may express concerns early on, while some children with autism may exhibit subtle features in early life and remain undiagnosed until after the age of three (Sacrey et al., 2018).

### The Psychometric Properties of the ADOS-2

The third aim focused on the predictive validity of one of the most commonly used diagnostic instruments for autism, the ADOS-2, in a sample of EL-siblings. The agreement between the ADOS-2 classifications and a later CBE diagnosis was found to be fair at both timepoints. While sensitivity and specificity were comparable at 14 and 24 months, both indicators were poor or marginally fair, suggesting a moderate ability to identify EL-children with a later CBE of autism.

These results indicate that a diagnosis of autism based on the ADOS-2 before the age of three is challenging in EL-siblings, where other developmental difficulties are highly prevalent (Charman et al., 2023). The sensitivity and specificity we observed are less than those reported in the review study by Lebersfeld et al. (2021). One possible explanation is that most of the included studies were carried out in a clinical setting, involving children who were referred after screening or for whom caregivers already had concerns, and in which the prevalence of autism was thus likely to be higher than in the current sample. Similar to the Q-CHAT, it is plausible that certain characteristics may become more evident and identifiable with the ADOS-2 at a later stage (Ozonoff et al., 2018; Shephard et al., 2017), potentially leading to a relatively high false negative rate at younger ages. It may also be that a subset of the children with a false-negative result has relatively strong cognitive abilities, compensating certain characteristics (Ozonoff et al., 2018). These children might develop coping mechanisms or adaptive behaviors that mask their autistic traits during early assessments, resulting in a false-negative outcome (Saban-Bezalel et al., 2022). Furthermore, parents of siblings may already have been involved in parent-mediated interventions, which could impact their interactions with their younger children (Zwaigenbaum et al., 2007). For instance, parents might adapt their interaction style based on their experiences with their autistic child, possibly providing more support. This altered interaction might mask some autistic traits in the younger siblings, contributing to false-negative results. Such adaptations could introduce a bias in parents’ observations and reporting, as they might perceive their younger child’s behaviour through the lens of their experiences with their older autistic child, potentially underreporting early signs of autism. Although this cannot be taken for granted, notable differences have been observed in mothers’ interactive behavior towards their autistic child compared to the younger sibling on certain aspects (Meirsschaut et al., 2011). A reasonable proportion of children also received a false positive result. While some children may exhibit behaviors pointing towards autism according to the ADOS-2, these may not be apparent enough in other assessments and contexts that are also considered in the diagnostic process. Previous studies already showed that some siblings exhibit subclinical features of autism, but fall short to meet the DSM-5 criteria for a diagnosis of autism (Charman et al., 2017; Tunç et al., 2021). These children with subclinical features may have contributed to the relatively high rate of false positives. In some cases, subthreshold autism traits may not pose challenges until later childhood, when the demands of the environment become too high for the individual, resulting in a so-called late onset diagnosis (Brian et al., 2016; Ozonoff et al., 2018; Shephard et al., 2017). This is an important consideration, as previous research with EL-siblings sometimes used 24 months as diagnostic outcome point (Shen et al., 2013; Wolff et al., 2014), while the current study follows children to 36 months of age and is thus better equipped to diagnose those with later onset of autism. Another potential explanation is that certain children receive a positive ADOS-2 result driven by social communication difficulties, yet a diagnosis of autism requires the presence of both social communicative difficulties and restricted, repetitive behaviors and interests, which they might not show. Lastly, EL-siblings may mimic certain behaviours from their older sibling or have less opportunity to develop social skills within sibling interaction (Bontinck et al., 2018). A follow-up study could investigate whether these early false positives on the ADOS-2 ultimately receive an autism diagnosis later in childhood.

In addition to examining sensitivity and specificity, we also evaluated the predictive validity of the ADOS-2 for providing an accurate classification of the presence or absence of autism in a particular child by means of predictive values. NPV was good, which suggests that the ADOS-2 is reasonably effective in accurately identifying children who do not have a CBE of autism. On the contrary, PPV was poor at all ages: the probability that EL-siblings with a positive ADOS-result at 14 or 24 months received a CBE diagnosis of autism at 36 months was lower than 50%. These observations highlight the complexity of diagnosing autism in EL-siblings and acknowledge that while some children may exhibit behaviours consistent with autism according to certain assessments like the ADOS-2, a comprehensive evaluation involving multiple instruments and informants is necessary to arrive at a clinical diagnosis of autism. Depending on other characteristics in these children, such as IQ and adaptive functioning, clinicians may be more or less inclined to diagnose autism.

### Clinical Implications

Screeners with good psychometric qualities could assist in identifying children with autism at an early age, when initial concerns arise. This could facilitate prompt referral, benefiting the child’s well-being, and potentially alleviating the burden of inaccurate referrals, thereby reducing pressure on waiting lists for multidisciplinary assessment (Palmer et al., 2011). While universal screening in the general population might not be advantageous (Siu, 2016), in EL siblings, where the prevalence of autism is higher, the benefits may outweigh the costs.

The trade-off between sensitivity and specificity, evident in both the Q-CHAT and the ADOS-2, emphasizes the challenges faced in EL-sibling cohorts. If the objective of screening purposes is to identify as many children as possible who may need further autism specific diagnostic assessment, high sensitivity is preferred. Nevertheless, it is equally important to minimize false positives to avoid waste of resources and avoid unjustified family worries. Although frequent early screening is often recommended (DeLucia et al., 2022; Johnson et al., 2007; Zwaigenbaum et al., 2015), our findings do not support this view. Our results suggest that the Q-CHAT is not suitable as a stand-alone screening instrument, primarily due to insufficient sensitivity, indicating a potential limitation in capturing early signs of autism in EL-siblings. Despite this, the high specificity indicates fairly well accuracy in identifying EL-siblings without autism. However, the question remains whether high specificity is desired considering that, in early childhood, emerging symptoms of autism often overlap with those observed in other groups of children who would also benefit from a more comprehensive assessment and intervention, such as children with general developmental delay or language impairment (Charman et al., 2016; Reiersen et al., 2007).

Regarding the usefulness of this instrument in providing an accurate indication of the presence or absence of autism in a particular child, our results emphasize the effectiveness of the Q-CHAT in correctly identifying children who do not need further autism specific clinical assessment (fair to good NPV estimates). The proportion of true positives leaves room for improvement, especially at 14 months, suggesting that clinicians should proceed with caution and refrain from screening too early with the Q-CHAT. In other words, relying on this screener for further referral could be adverse to children and their families due to the potential stress of unnecessary referrals.

Concerning diagnostic assessment with the emphasis on identifying true positives with high specificity, our findings pose challenges. The ADOS-2 demonstrates a moderate balance between sensitivity and specificity resulting in a high false positive rate in this population. Furthermore, the consistently modest PPV observed across both ages necessitates a nuanced interpretation of positive diagnostic outcomes. These findings confirm the critical need not to depend exclusively on the ADOS-2 for autism assessments. This approach reflects the deliberate practice among clinicians who do not base diagnoses solely on ADOS classifications. Therefore, recognizing that autism is more complex than what is measured by the ADOS alone requires consideration of the broader array of factors influencing clinical decisions, such as familial history and clinician judgment. A more comprehensive multidisciplinary approach, incorporating clinical judgment and additional evaluations, remains crucial.

### Strengths and Limitations

This study has several strengths, including a large number of EL-children, data collected at three important developmental timepoints and the inclusion of children without CBE of autism. However, it also has some limitations. Firstly, the study involved highly educated and voluntary participants, which limits the generalizability. Secondly, there is a large amount of missing data at 14 months. However, there were fewer missing data at 24 months and the children did meet the criteria to administer an ADOS at that time, but the psychometric qualities did not improve. Thirdly, the validity of the ADOS-2 assessment at 14 months might be affected because not all children were able to walk and some children had a developmental age below 12 months. Fourthly, we recognise that the sensitivity, specificity, PPV, and NPV estimates presented in this study are influenced by the limitations inherent in our sample, such as potential selection bias and missing data, as well as the absence of a perfect gold standard. Given that these results are derived from a single study, caution is advised in interpreting these metrics. Replication of these findings in larger, more diverse samples is necessary to strengthen their generalisability and confirm their robustness. Furthermore, there may be differences between CBE diagnoses and clinical diagnoses. Although these are largely determined in the same manner, we cannot be certain that they correspond perfectly. Consequently the use of a research diagnosis rather than a clinical diagnosis in our study may affect the reliability, validity, and general applicability of our results. Additionally, we did not take into account possible interventions that children or their parents received, which could potentially influence autism characteristics. Finally, follow-up until the age of 36 months may not provide a complete picture. Future studies with longer follow-up periods are needed to understand the developmental trajectories and long-term outcomes.

## Conclusion

In conclusion, the Q-CHAT can be helpful in making an early distinction between EL-children who require further diagnostic assessment and those who do not, primarily by excluding children who do clearly not have autism. However, our data do not support relying on the Q-CHAT as standalone screening instrument, even in EL-siblings where parents may be more aware of autism features. Low sensitivity and low PPV are a limiting factor. The ADOS-2 at a young age provides useful information about the presence of autism characteristics in EL-siblings, but is insufficient on its own to distinguish between children who will or will not receive a diagnosis later on.
